# Supplementary material for: Bactericidal Activity of Non-Cytotoxic Cationic Nanoparticles against Clinically and Environmentally Relevant Pseudomonas spp. Isolates
Source: Pharmaceutics. 2021 Sep 6;13(9):1411. doi: 10.3390/pharmaceutics13091411 (PMC8465415; doi:10.3390/pharmaceutics13091411)
Supplement: Supplementary file 1 [file pharmaceutics-13-01411-s001.zip › pharmaceutics-1357671-supplementary.pdf]

Supplementary Material

Bactericidal Activity of Non-Cytotoxic Cationic Nanoparticles Against Clinically and Environmentally Relevant *Pseudomonas* spp. Isolates

Anna Maria Schito [1], Gabriella Piatti [1], Debora Caviglia [1], Guendalina Zuccari [2] Alessia Zorzoli [3], Danilo Marimpietri [3] and Silvana Alfei [2,*]

[1] Department of Surgical Sciences and Integrated Diagnostics (DISC), University of Genoa, Viale Benedetto XV, 6, I-16132 Genova, Italy; amschito@unige.it (A.M.S), gabriella.piatti@unige.it (G.P.), Cavigliad86@gmail.com (D.C.)
[2] Department of Pharmacy, University of Genoa, Viale Cembrano, 16148 Genoa, Italy; zuccari@difar.unige.it (G.Z.)
[3] Laboratory of Stem Cell and Cell Therapy, IRCCS Istituto G. Gaslini via Gerolamo Gaslini 5 16147 Genova Italy; alessiazorzoli@gaslini.org (A.Z.), danilomarimpietri@gaslini.org (DM)
* Correspondence: alfei@difar.unige.it; Tel.: +39 010 355 2296 (S.A.)

Section S1. Synthesis of dendrimer G5-PDK [1-6].

S1.1. Synthesis of the Uncharged Fifth Generation Inner Scaffold of G5-PDK (G5-PD-OH)

Performing previously reported procedures [2-6], starting from the AB_2 monomer known as *bis*-hydroxymethyl propanoic acid *bis*-HMPA, firstly, we prepared the fifth generation dendron D5-A-COOH (Figure S1), and then, according to Scheme S1, we synthetized the uncharged dendrimer G5-PD-OH [4].

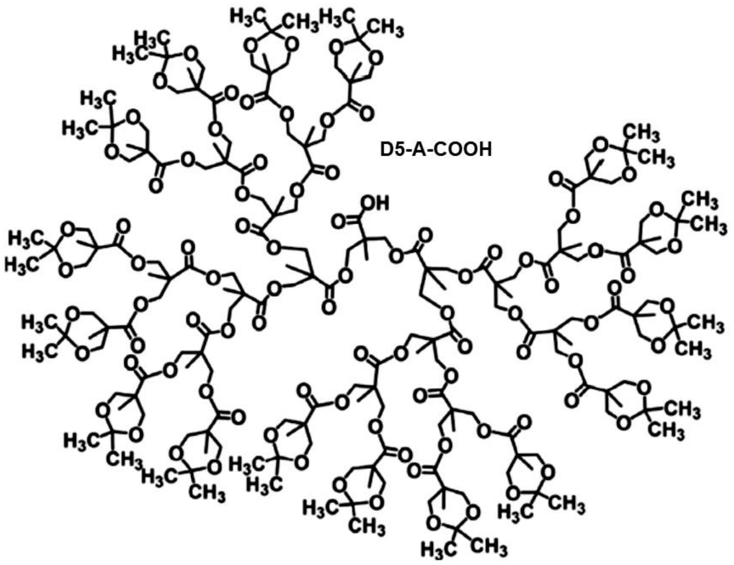

Figure S1. Structure of dendron intermediate (D5-A-COOH), prepared to synthetize dendrimer G5-PD-A.

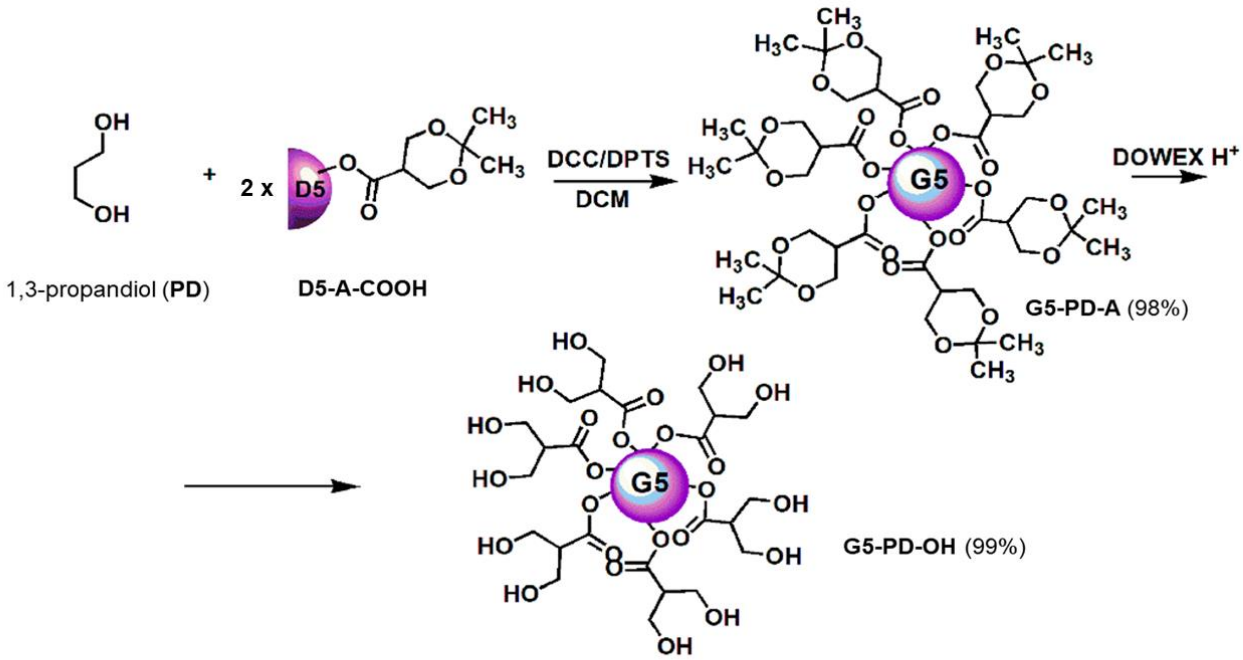

Scheme S1. Synthetic procedure for achieving dendrimer G5-PD-OH. D = Dendron; A = acetonide protected; COOH = free carboxylic group; D5 = generations number of dendron; G5 = generations number of dendrimers; PD = propanediol; DCC = *N,N′*-dicyclohexylcarbodiimide; DPTS = 4-(dimethyl-amino)pyridinium 4-toluene-sulfonate; DCM = dichloromethane; DOWEX H^+ = acid resins.

S1.1.1. FTIR, NMR spectral data and Elemental analysis results of G5-PD-OH [4]

FTIR (KBr, cm^{-1}): 3436 (OH), 2936, 1737 (C=OO). ^1H NMR (400 MHz, DMSO-*d6*), δ (ppm): 1.01, 1.16, 1.18, 1.23, 1.34 (five s signals, 186H, CH_3 of generations), 1.70 (m, 2H, CH_2 propanediol), 3.52 (dd, 128H, CH_2OH), 3.56 (partially overlapped signal, 2H, CH_2O propanediol), 3.98 (partially overlapped signal, 2H, CH_2O propanediol), 4.08-4.18 (m, 120H, CH_2O of four generations), 4.37 (br s, 64H, OH). ^{13}C NMR (100 MHz, DMSO-*d6*), δ (ppm): 173.94, 171.73 (C=O), 64.27, 63.55 (CH_2O), 50.13 (quaternary C of fifth generation), 46.12 (other generations detectable quaternary C), 17.05, 16.61 (CH_3 of generations). Found: C, 51.71; H, 7.01. $C_{313}H_{504}O_{188}$ requires C, 51.67; H, 6.98%.

S1.2. Synthesis of Lysine-Modified Cationic Dendrimer G5-PDK (128 HCl) [1]

S1.2.1. Synthesis of Lysine-Modified Boc-Protected Dendrimer G5-PD-BK

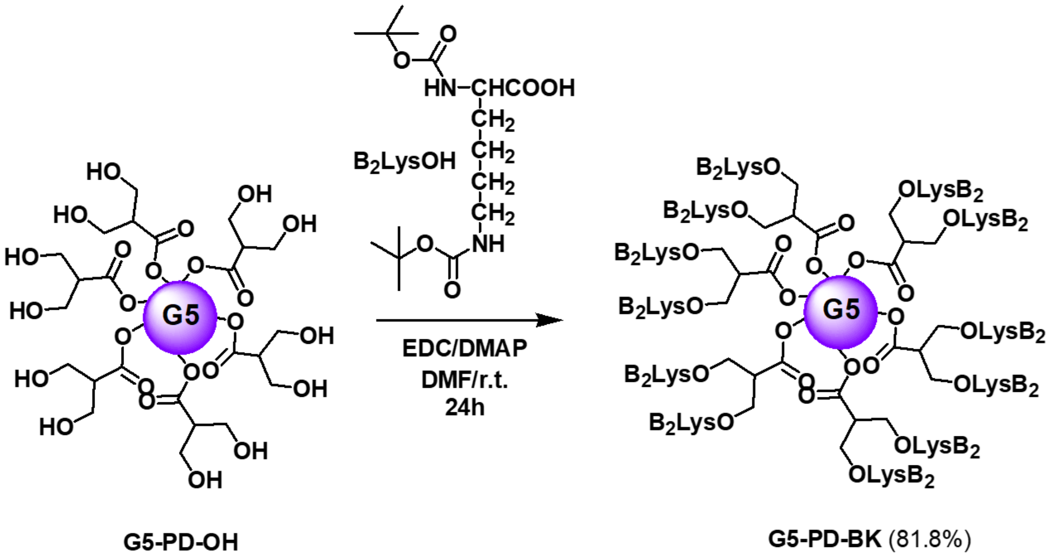

Scheme S2. Synthesis of Boc-protected dendrimer G5-PD-BK. PD = propanediol; B = Boc-protecting group; Lys or K = Lysine; G5 = generations number [1].

A solution of G5-PD-OH (68.3 mg; 0.0094 mmol) in dry DMF (1.5 mL) was added with Boc_2-Lys-OH (76.8 equiv; 250.0 mg; 0.7209 mmol), 4-dimethylaminopyridine (DMAP) (38.4 equiv.; 44.1 mg; 0.3610) and *N*-ethyl-*N*-(3-dimethylamino)propyl carbodiimide hydrochloride (EDC) (76.8 equiv.; 111.9 mg; 0.7209 mmol). The solution was kept under magnetic stirring at r.t. for 24 h then added with 15 mL of ethyl acetate (EtOAc) to produce a suspension which was washed with 10% aq. $KHSO_4$ (3x15 mL). The aqueous washings were extracted with EtOAc and the combined organic phases were washed with aq. 15% NaOH followed by water then dried on $MgSO_4$ overnight. The removal of the solvent at reduced pressure afforded the Boc-protected lysine-modified dendrimer G5-PD-BK as off white glassy solid (217.5 mg; 0.0077 mmol; 81.8% yield).

FTIR (KBr, cm^{-1}): 3380 (NH), 1747 (C=O ester), 1710 (C=O urethane), 1527 (NH). ^{1}H NMR (400 MHz, $CDCl_3$), δ (ppm): 0.95-1.90 (m, 572H, CH_3 of dendrimer + CH_2O propanediol + $CH_2CH_2CH_2$ of Lys), 1.43 (s, 576H, CH_3 of Boc), 1.44 (s, 576H, CH_3 of Boc), 3.10 (m, 128H, CH_2NH of Lys), 3.56 (partially overlapped signal, 2H, CH_2O propanediol), 4.25 (m, 314H, CH_2O of dendrimer and of propanediol + CHNH of Lys), 4.70-5.50 (m, 128H, $^{\alpha}$NHBoc + $^{\varepsilon}$NHBoc of Lys). ^{13}C NMR ($CDCl_3$, 100 MHz), δ (ppm): 14.20-17.90 (CH_3 of G1, G2, G3, G4, G5), 22.57 (CH_2), 28.36 (CH_3 of Boc), 28.47 (CH_3 of Boc), 29.57 (CH_2), 31.84 (CH_2), 40.04 (CH_2NH), 46.42 (quaternary C), 53.37 (CHNH), 65.41-65.60 (CH_2O of G1, G2, G3, G4, G5), 79.02 (quaternary C of Boc), 79.80 (quaternary C of Boc), 155.63 (C=O urethane), 156.17 (C=O urethane), 172.32 (C=O amino acid + C=O ester of G1, G2, G3, G4, G5), CH_2 of propanediol not detectable. Found: C, 56.78; H, 8.30; N, 6.00. $C_{1337}H_{2296}N_{128}O_{508}$ requires C, 56.76; H, 8.18; N, 6.34%.

S1.2.2. Acidic Deprotection of G5-PD-BK to obtain G5-PDK * 128 HCl

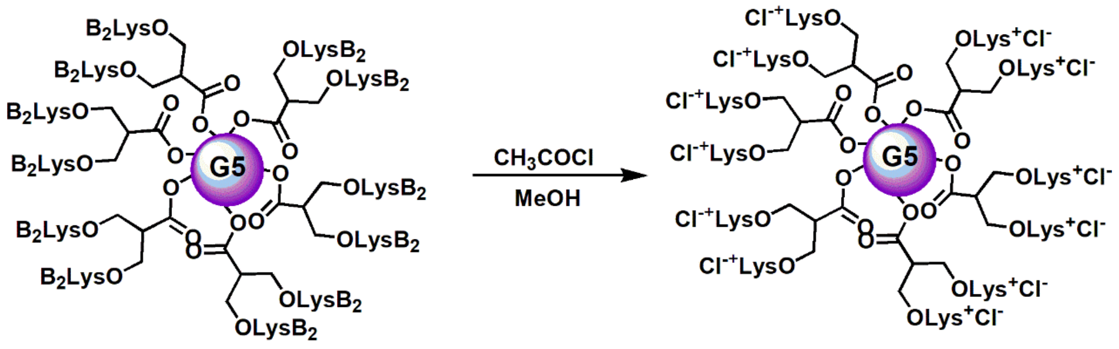

G5-PD-BK (81.8%) **G5-PDK*HCl** (99%)

Scheme S3. Synthesis of cationic dendrimer G5-PDK having 128 protonated nitrogen atoms. PD = propanediol; B = Boc-protecting group; Lys or K = Lysine; G5 = generations number [1].

A solution of G5-PD-BK (208.8 mg; 0.0074 mmol) in 1 mL methanol (MeOH) was cooled to 0 °C and treated with acetyl chloride (2 equiv./Boc-groups to be removed; 148.3 mg; 1.8893 mmol; 134.8 µL). The solution was kept at r.t. under magnetic stirring for 24 h, then it was concentrated at reduced pressure, taken with MeOH, and precipitated into acetone. The dendrimer in the form of hydrochloride was recovered as oil after centrifugation, washed repeatedly with fresh acetone, recovered all times by centrifugation, and finally dried at reduced pressure. G5-PDK * 128 HCl was obtained as white highly hygroscopic solid, which was stored under vacuum over P_2O_5. (147.2 mg, 0.0073 mmol, 99 % yield).
FTIR (KBr, cm^{-1}): 3431 (NH_3^+), 1744 (C=O ester), 1635 (NH). ^{1}H NMR (400 MHz, DMSO-*d6*), δ (ppm): 1.03-1.99 (m, 570H, CH_3 of dendrimer + $CH_2CH_2CH_2$ of Lys), 1.70 (m, 2H, CH_2 propanediol), 2.76 (m, 128H, $CH_2NH_3^+$ of Lys), 3.56 (partially overlapped signal, 2H, CH_2O propanediol), 3.99 (m, 64H, $CHNH_3^+$ of Lys), 4.10-4.50 (m, 250H, CH_2O of propanediol and of dendrimer + $CHNH_3^+$ of Lys), 8.20 (br s,192H, $^{\alpha}NH_3^+$), 8.82 (br s, 192H, $^{\varepsilon}NH_3$+ of Lys). ^{13}C NMR (DMSO-*d6*, 100 MHz), δ (ppm): 19.33 (CH_3), 23.14 (CH_2), 28.01 (CH_2), 31.01 (CH_2), 40.02 ($CH_2NH_3^+$), 47.70 (quaternary C), 53.55 ($CHNH_3^+$), 67.65-67.82 (CH_2O and of G1, G2, G3, G4), 170.68-173.33 (C=O of amino acid + ester of G1, G2, G3, G4), CH_2 of propanediol not detectable. Found: C, 56.78; H, 8.30; N, 6.00. $C_{697}H_{1400}N_{128}O_{252}Cl_{128}$ requires C, 41.56; H, 7.00; N, 8.90; Cl, 22.53%.

Section S1.2.3. Potentiometric titration of G5-PDK [1].

Table S1. Data of potentiometric titration used for constructing the titration curve and those of dpH/dV used for constructing the first derivative curve.

mL HCl 0.1N	pH	dpH/dV
0.0	9.54 ± 0.02	---
0.2	9.30 ± 0.03	1.2
0.4	9.00 ± 0.02	1.5
0.6	6.85 ± 0.05	10.75
0.8	6.15 ± 0.01	3.5
1.0	5.60 ± 0.04	2.75
1.2	4.80 ± 0.02	4
1.4	4.65 ± 0.02	0.75
1.6	4.50 ± 0.03	0.75
1.8	4.45 ± 0.02	0.25
2.0	4.40 ± 0.04	0.25
2.2	4.35 ± 0.02	0.25
2.4	4.30 ± 0.01	0.25
2.6	4.30 ± 0.009	0
2.8	4.20 ± 0.009	0.5
3.0	4.15 ± 0.01	0.25
	**G5-PDK**	
Max dpH/dV	10.75	4.0
HCl (mL)	0.6	1.2
pH	6.85	4.80

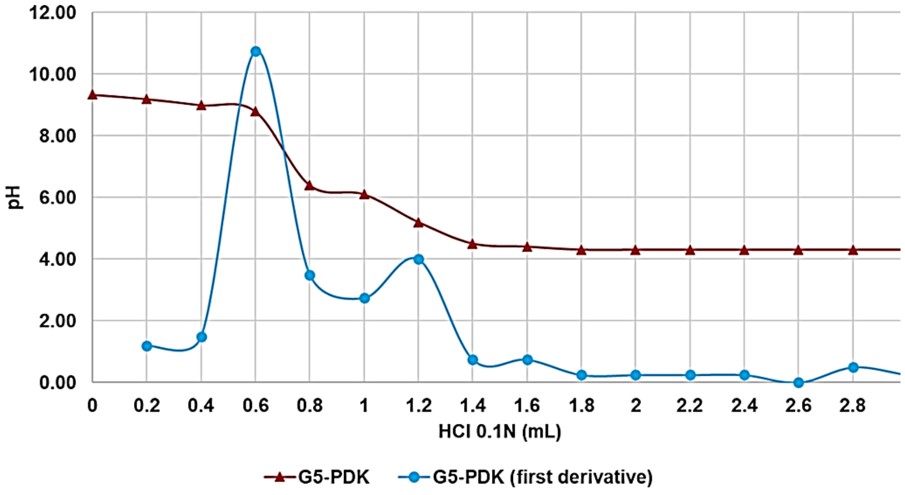

Figure S2. Titration curve of G5-PDK (red line); first derivative line of the titration curve (light blue line), which shows separate peaks in correspondence of each end point of the titration curve [1].

Table S2. Main physical features of G5-PDK [1].

Physical characteristics	G5-PDK	
N[1]	128	
MW (calc.) [2]	20145.3	
MW (obs.) [3]	19961.2±480.2	
Error (%)	-0.9%	
Z-Ave (nm) [4]	203.0±2.6 [5]	203.0±2.6 [6]
PDI [7]	0.282±0.028 [5]	0.282±0.028 [6]
ζ-p (mV) [8]	+19.2±7.3	

[1] Number of protonated nitrogen atoms; [2] molecular weight (MW) computed according to the structure of G5-PDK confirmed by NMR analysis and elemental analysis; [3] MW obtained by volumetric titration; [4] particle size by dynamic light scattering (DLS) analysis; [5] intensity-weighted mean hydrodynamic diameters; [6] number-weighted mean hydrodynamic diameters; [7] polydispersity index (DLS); [8] Z-potential (DLS).

Section S2. Antibacterial properties of G5-PDK evaluated against the most relevant MDR representative of both Gram-negative and Gram-positive species [1].

Table S3. MIC values of G5-PDK on relevant representatives of Gram-positive and Gram-negative bacteria obtained from experiments carried out in triplicate[1], expressed as µM and as µg/mL.

	**G5-PDK (20145)[2]**	**Ciprofloxacin**
**Strains**	**MIC** µM (µg/mL)	**MIC** µM (µg/mL)
E. faecalis *	>25.4 (>512)	193.2 (64)
E. faecium *	>25.4 (>512)	772.7 (256)
S. aureus **	>25.4 (>512)	386.4 (128)
S. epidermidis **	>25.4 (>512)	193.2 (64)
E. coli #	>25.4 (>512)	96.6 (32)
K. pneumoniae #	>25.4 (>512)	96.6 (32)
A. baumannii	6.3 (128)	193.2 (64)

[1] The degree of concordance was in all the experiments 3/3, and standard deviation (±S.D.) was zero; [2] MW of G5-PDK; * denotes vancomycin resistant isolates (VRE); ** denotes methicillin resistant isolates; # denotes a carbapenemase (KPC)-producing bacterium; *A. baumannii* was a MDR strain.

Table S4. MICs of G5-PDK obtained on isolates of the genus *Acinetobacter* from experiments conducted in triplicate [1] (expressed as µM and as µg/mL) compared to the MICs of ciprofloxacin.

**G5-PDK (20145)[2]**		**Ciprofloxacin**
**Strains**	**MIC** µM (µg/mL)	**MIC** µM (µg/mL)
A. baumannii 236	6.3 (128)	193.2 (64)
A. baumannii 245	6.3 (128)	1.6 (0,5)
A. baumannii 257	6.3 (128)	96.6 (32)
A. baumannii 279	12.7 (256)	48.3 (16)
A. baumannii 383	6.3 (128)	193.2 (64)
A. baumannii 406	6.3 (128)	96.6 (32)
A. johnsonii 387	6.3 (128)	0.9 (0.3)
A. junii 389	12.7 (256)	0.4 (0.125)
A. pittii 263	6.3 (128)	3.2 (1)
A. pittii 272	6.3 (128)	1.6 (0.5)
A. ursingii 388	3.2 (64)	0.4 (0.125)
A. ursingii 408	6.3 (128)	0.8 (0.25)

[1] The degree of concordance was in all the experiments 3/3, and S.D. was zero; [2] MW of G5-PDK; *Acinetobacters* are all MDR bacteria.

Section S3. Cytotoxicity Experiments

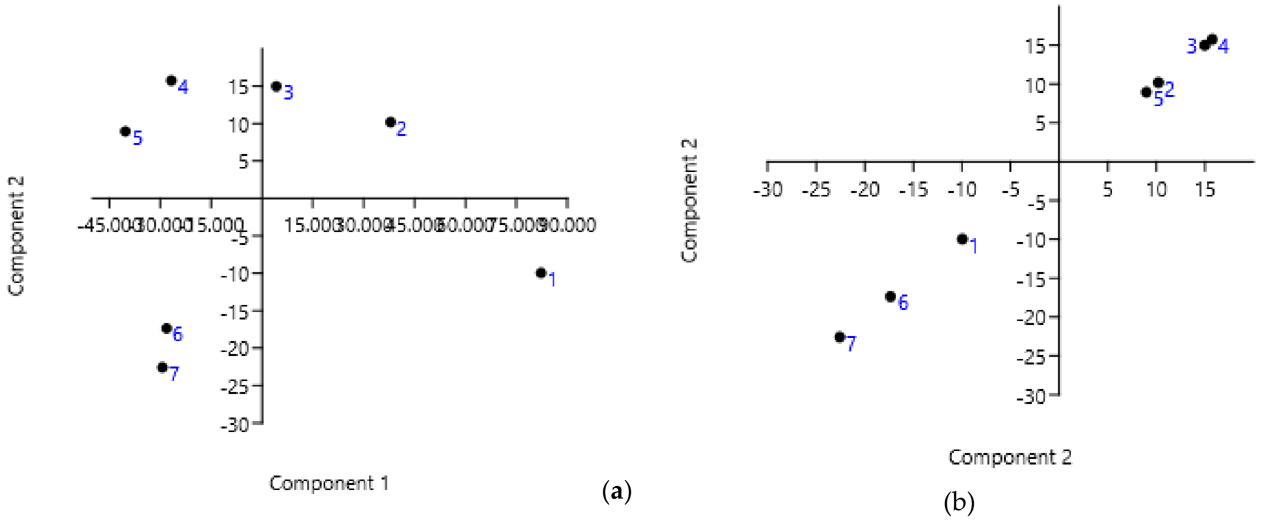

(**a**) (b)

Figure S3. PCA results on data concerning 12 h of cells exposure: score plot showing data locations on PC1 vs PC2 (**a**); score plot showing data locations on PC2 vs PC2 (**b**).

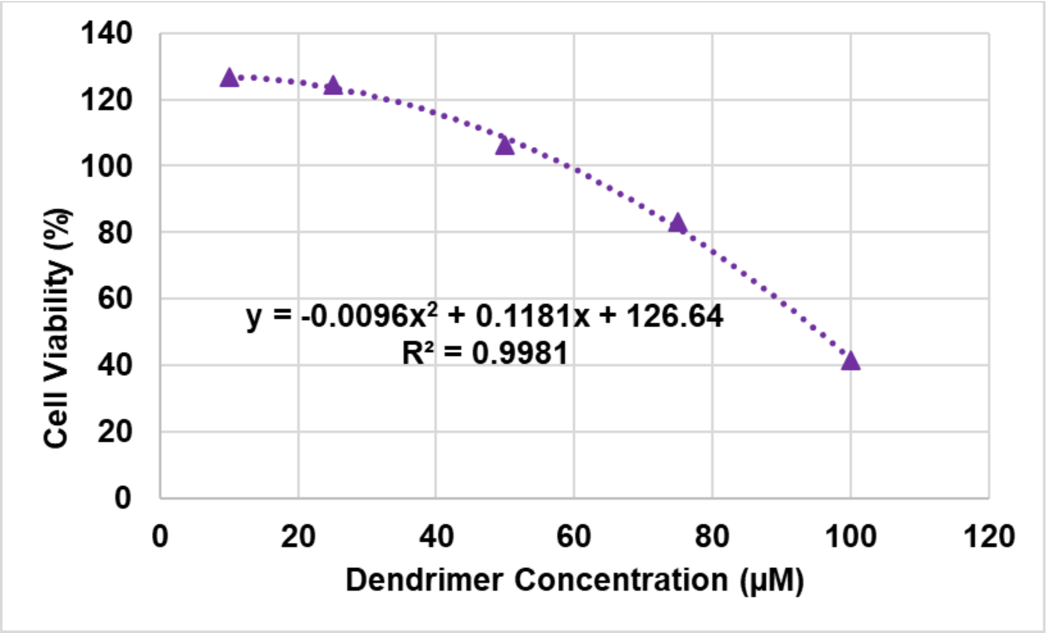

Figure S4. Second-degree polynomial regression model obtained from data concerning 12 h of cells exposure.

References

1. Alfei, S.; Caviglia, D.; Piatti, G.; Zuccari, G.; Schito, A.M. Bactericidal Activity of a Self-Biodegradable Lysine-Containing Dendrimer against Clinical Isolates of *Acinetobacter* Genus. *Int. J. Mol. Sci.* **2021**, *22*, 7274. https://doi.org/10.3390/ijms22147274.
2. Ihre, H.; Hult, A.; Fréchet, J.M.J.; Gitsov, I. Double-stage convergent approach for the synthesis of functionalized dendritic aliphatic polyesters based on 2,2-bis(hydroxymethyl)propionic acid. *Macromolecules* **1998**, *31*, 4061–4068.

3. Alfei, S.; Castellaro, S.; Taptue, G.B. Synthesis and NMR characterization of dendrimers based on 2,2-bis-(hydroxymethyl)-propanoic acid (bis-HMPA) containing peripheral amino acid residues for gene transfection. *Org. Commun.* **2017**, *10*, 144–177.
4. Alfei, S.; Castellaro, S. Synthesis and characterization of polyesterbased dendrimers containing peripheral arginine or mixed amino acids as potential vectors for gene and drug delivery. *Macromol. Res.* **2017**, *25*, 1172–1186.
5. Alfei, S.; Catena, S.; Turrini, F. Biodegradable and biocompatible spherical dendrimer nanoparticles with a gallic acid shell and a double-acting strong antioxidant activity as potential device to fight diseases from "oxidative stress". *Drug Deliv. Transl. Res.* **2019**, 1–12.
6. Alfei, S.; Signorello, M.G.; Schito, A.M.; Catena, S.; Turrini, F. Reshaped as polyester-based nanoparticles, gallic acid inhibits platelet aggregation, reactive oxygen species production and multi-resistant Gram-positive bacteria with an efficiency never obtained. *Nanoscale Adv.* **2019**, *1*, 4148–4157.
